# Cognitive Impairment in Cancer Patients Treated with Immune Checkpoint Inhibitors: A Cross-Sectional Study

**DOI:** 10.3390/jcm15020689

**Published:** 2026-01-15

**Authors:** Betul Aktepe, Oktay Halit Aktepe

**Affiliations:** 1 Department of Psychiatry, Urla State Hospital, Ministry of Health, Izmir 35430, Türkiye; bdabakolu@gmail.com; 2 Department of Medical Oncology, Dokuz Eylul University, Izmir 35330, Türkiye

**Keywords:** cancer, cognitive impairment, immune checkpoint inhibitors, MoCA, quality of life

## Abstract

**Background and Objectives:** Immune checkpoint inhibitors (ICIs) have transformed cancer care, but their impact on cognition is unclear. This study examined the prevalence and clinical correlates of cognitive impairment in patients receiving ICIs. **Materials and Methods:** In this two-center, cross-sectional cohort of 189 adults with solid tumors treated with ICIs, cognitive function was assessed using the Montreal Cognitive Assessment (MoCA). Cognitive impairment was defined as MoCA ≤ 21. Age, sex, education, Eastern Cooperative Oncology Group (ECOG) performance status, treatment line, number of metastatic sites, and ICI exposure were compared between cognitive groups using chi-square tests. Univariate and multivariate logistic regression models were used to identify independent predictors of cognitive impairment. **Results:** The median age was 65 years and 73% of patients were male. Overall, 102 of 189 participants (54%) met criteria for cognitive impairment. Patients with impaired cognition were more often aged ≥65 years, female, and educated at or below high school level, and more frequently had ECOG ≥ 1, second- or later-line ICI therapy, and ≥2 metastatic sites (all *p* < 0.05). In multivariate analysis, independent predictors of cognitive impairment were age ≥ 65 years (OR: 2.98, 95% CI 1.45–6.12, *p* = 0.003), female sex (OR: 2.48, 1.09–5.67, *p* = 0.030), lower education (OR: 3.10, 1.35–7.07, *p* = 0.007), later-line therapy (OR: 3.51, 1.56–7.88, *p* = 0.002), ECOG ≥ 1 (OR: 3.38, 1.46–7.83, *p* = 0.004), and ≥2 metastatic sites (OR: 2.85, 1.37–5.90, *p* = 0.005). **Conclusions:** More than half of patients receiving ICIs exhibit objective cognitive deficits. Systematic cognitive screening in high-risk subgroups may allow for earlier recognition of impairment and more timely supportive care.

## 1. Introduction

Immune checkpoint inhibitors (ICIs) have markedly changed the treatment of various cancers, extending survival outcomes even in metastatic settings [1]. As ICIs become widely integrated into treatment for various tumor types, the association between ICI therapy and cognitive impairment has yet to be clearly defined. Chemotherapy is a well-established cause of cognitive impairment, known as cancer-related cognitive impairment (CRCI), typically involving attention, memory, and executive deficits, with reported rates varying between 15% and 75% [2,3]. These cognitive problems can substantially impair daily functioning and quality of life (QoL) [4]. In contrast, the cognitive effects of ICIs are still not well defined [5]. Neurological immune-related adverse events, including hypophysitis and encephalitis, are uncommon but well-recognized complications of ICIs [6]. It has been suggested that ICIs may trigger subtle neuroinflammation or subclinical encephalopathy, which impairs cognition without obvious neurologic symptoms [7,8,9]. ICI-related immune cell activation and cytokine release have been shown to impair the blood–brain barrier and cause neuronal injury [10], providing a biological rationale for potential cognitive changes. However, cognitive changes observed during ICI therapy may stem largely from previous chemotherapy exposure rather than from the effects of ICIs themselves [5].

Another important consideration is which patients might be most vulnerable to ICI-related cognitive effects. Previous studies on CRCI have identified several risk factors, including older age [11], female sex [12], and lower educational attainment [13,14]. Additionally, patients with advanced or metastatic disease tend to experience greater cognitive impairment than those with localized tumors [12]. Together, these factors highlight that vulnerability to cognitive impairment during ICI therapy may be influenced by a combination of patient-related and disease-related characteristics rather than treatment exposure alone. In addition, unrecognized cognitive impairment may negatively impact treatment adherence, reliable symptom reporting, and informed decision-making. Identifying patients at higher risk may facilitate earlier recognition of cognitive difficulties and support more individualized monitoring and supportive care strategies during ICI therapy. Therefore, the present study aimed to investigate cognitive function in cancer patients receiving ICIs and to explore associations with the clinical variables. We utilized the Montreal Cognitive Assessment (MoCA) as an objective measure due to its high sensitivity for detecting mild cognitive impairment [15].

## 2. Materials and Methods

### 2.1. Participants and Study Design

This two-center, cross-sectional study was conducted between October 2022 and October 2025 at the Departments of Medical Oncology of Dokuz Eylul University (Izmir, Türkiye) and Bolu Abant Izzet Baysal University (Bolu, Türkiye). Eligible participants were adult patients (≥18 years) with histologically confirmed solid tumors who were receiving ICIs, including Programmed cell death protein-1 (PD-1), anti–Programmed death-ligand-1 (PD-L1), or anti–Cytotoxic T-lymphocyte–associated protein-4 (CTLA-4) antibodies, either as monotherapy or combination regimens. All patients had an Eastern Cooperative Oncology Group (ECOG) performance status of 0 to 2. Patients were required to have completed at least three cycles of ICI therapy prior to cognitive testing. Exclusion criteria included (i) known central nervous system (CNS) metastases, (ii) prior cranial radiotherapy within the last six months, (iii) history of neurodegenerative or severe psychiatric disorders, (iv) current corticosteroid use ≥ 10 mg prednisone equivalent daily, and (v) inability to complete cognitive testing due to language or sensory limitations. Patients who provided written informed consent were recruited consecutively from the outpatient oncology clinics of the participating centers during the study period, based on predefined eligibility criteria.

Baseline demographic and clinical data were obtained from patient interviews and electronic medical records. Recorded variables included age, sex, marital and educational status, cancer type, disease stage, ECOG performance status, number and sites of metastases, line of ICIs, number of ICI cycles, and comorbidities, including hypertension (HT), diabetes mellitus (DM), cardiovascular disease (CVD). Educational attainment was categorized as ≤high school versus ≥university, reflecting cognitive reserve levels. Disease burden was classified by the number of metastatic sites (<2 vs. ≥2), and treatment exposure was grouped by cycle number.

### 2.2. Assessment of Cognitive Function

Cognitive performance was evaluated using the MoCA, a validated screening instrument widely applied to detect mild cognitive impairment in oncology and geriatric populations [16,17]. The Turkish version of the test, which has been established as reliable and valid [18], was administered by trained physicians and oncology nurses in a quiet and distraction-free clinical setting. Assessors did not participate in treatment decisions and were blinded to detailed clinical information beyond basic eligibility information. Assessments were performed within two days after the scheduled ICI infusion, including the infusion day. The standard 1-point correction was applied for participants with ≤12 years of education. Based on prior studies in cancer cohorts, a MoCA total score of ≤21 was defined as cognitive impairment, while scores > 21 were considered normal. The total administration time per participant was approximately 10–15 min.

### 2.3. Statistical Analysis

The primary outcome of this study was the prevalence of cognitive impairment among patients receiving ICIs, as assessed by the MoCA. Secondary outcomes included associations between cognitive status and key clinical-demographic factors such as age, sex, education level, ECOG performance status, number of metastatic sites, treatment line, and number of ICI cycles. Given the exploratory, cross-sectional nature of the study and its primary aim of estimating prevalence and associations, no a priori sample size calculation or formal power analysis was performed. The sample size was determined by the number of eligible patients who consented to participate in the study during the study period.

Continuous variables were expressed as mean ± standard deviation (SD) or median (interquartile range, IQR), whereas categorical variables were summarized as frequencies and percentages. Comparisons between groups were performed using either the independent-samples *t* test or the Mann–Whitney U test for continuous data, while categorical variables were analyzed with the chi-square test or Fisher’s exact test, as appropriate. To explore potential independent predictors of cognitive impairment, all relevant variables were first evaluated using univariate logistic regression analyses. Variables with a *p* value < 0.10 in univariate analyses were subsequently included in a multivariable logistic regression model, with results reported as odds ratios (ORs) and corresponding 95% confidence intervals (CIs). All statistical analyses were performed using IBM SPSS Statistics version 27 (IBM Corp., Armonk, NY, USA), with two-sided *p* values < 0.05 considered indicative of statistical significance.

## 3. Results

### 3.1. Baseline Clinical and Demographic Characteristics

A total of 189 patients who received ICIs were included in the analysis. Clinical and demographic characteristics of the study cohort, grouped by MoCA-based cognitive status, are presented in Table 1. The median age of the cohort was 65 years, with 54.5% of the participants being younger than 65. Most patients were male (73%) and had an ECOG performance status of 1–2 (63.5%). The majority had non-small cell lung cancer (NSCLC, 58.2%), followed by renal cell carcinoma (RCC, 13.8%), melanoma (9.5%), and other malignancies (18.5%). Regarding treatment details, 69.8% of patients received ICIs as second-line or later-line therapy, and 54% had completed ≥6 cycles at the time of cognitive assessment. Among the 189 patients, anti–PD-1 monotherapy was the predominant regimen (*n* = 158; nivolumab 142 [75.1%] and pembrolizumab 16 [8.5%]), while anti–PD-L1 agents were used less frequently (*n* = 25; atezolizumab 16 [8.5%], avelumab 6 [3.2%], durvalumab 3 [1.6%]), and anti–CTLA-4–based therapy was administered only in a small subset (*n* = 6 [3.2%]). HT, DM, and CVD were present in 25.9%, 10.2%, and 11.8% of patients, respectively.

In the overall cohort, the mean MoCA score was 20.6 ± 4.6, with a median of 20 (IQR: 17–25). MoCA scores ranged from 10 to 29. Among patients with normal cognition (MoCA > 21), the mean MoCA score was 24.9 ± 1.8, with a median of 25 (IQR: 24–26; range, 22–29). However, patients with cognitive impairment (MoCA ≤ 21) had a mean MoCA score of 16.9 ± 2.4, with a median of 17 (IQR: 15–19; range, 10–21). In accordance with the standard scoring procedure, a 1-point education correction was applied to 116 of 189 patients (61.4%). The median number of ICI cycles at assessment was 6 (IQR, 5–9). According to MoCA scores, 87 patients (46%) were classified as cognitively normal (MoCA > 21), whereas 102 patients (54%) demonstrated impaired cognition (MoCA ≤ 21). When stratified by treatment exposure, cognitive impairment was observed in 40.4% (23/57) of patients receiving first-line ICI monotherapy, compared with 59.8% (79/132) of those treated in second-line or beyond settings. Compared with cognitively normal patients, those with cognitive impairment were significantly older (≥65 years: 56.9% vs. 32.2%, *p* < 0.001) and more frequently female (34.3% vs. 18.4%, *p* = 0.014). Educational attainment also differed substantially between groups, as patients with a high school level or lower education were more prevalent among those with cognitive decline (79.4% vs. 40.2%, *p* < 0.001). Similarly, poor performance status was more frequent in the impaired group (ECOG ≥ 1: 80.4% vs. 43.7%, *p* < 0.001), indicating a strong relationship between functional status and cognitive outcomes. In contrast, primary tumor type, specific metastatic sites, and comorbid conditions such as HT, DM, or CVD did not significantly differ between groups (all *p* > 0.05). Nonetheless, a longer ICI treatment duration (≥6 cycles) was also associated with a higher prevalence of cognitive impairment compared with shorter treatment duration (<6 cycles) (63.7% vs. 42.5%, *p* = 0.004).

### 3.2. Factors Associated with Cognitive Impairment

As shown in Table 2, univariate and multivariate logistic regression analyses identified the factors associated with cognitive impairment. In univariate analyses, age ≥ 65 years (OR: 2.77, 95% CI 1.53–5.04, *p* < 0.001), female sex (OR: 2.31, 95% CI 1.17–4.57, *p* = 0.015), lower educational level (OR: 5.73, 95% CI 3.01–10.90, *p* < 0.001), second- or later-line therapy (OR: 2.20, 95% CI 1.17–4.15, *p* = 0.014), ECOG performance status ≥ 1 (OR: 5.28, 95% CI 2.76–10.09, *p* < 0.001), ≥2 metastatic sites (OR: 2.21, 95% CI 1.23–3.96, *p* = 0.008), and treatment duration ≥ 6 cycles (OR: 2.37, 95% CI 1.32–4.26, *p* = 0.004) were identified as significant predictors of cognitive impairment. In the multivariate model, independent predictors of poor cognitive status included older age (OR: 2.98, 95% CI 1.45–6.12, *p* = 0.003), female sex (OR: 2.48, 95% CI 1.09–5.67, *p* = 0.030), lower educational level (OR: 3.10, 95% CI 1.35–7.07, *p* = 0.007), later-line therapy (OR: 3.51, 95% CI 1.56–7.88, *p* = 0.002), poor performance status (ECOG ≥ 1; OR 3.38, 95% CI 1.46–7.83, *p* = 0.004), and the presence of ≥2 metastatic sites (OR: 2.85, 95% CI 1.37–5.90, *p* = 0.005). Although prolonged treatment exposure (≥6 cycles) was associated with cognitive decline in univariate analysis, it did not remain statistically significant after adjustment for confounding factors (*p* = 0.204).

## 4. Discussion

In this study, we found that cognitive impairment is a common issue in cancer patients receiving ICIs. As most patients received ≥6 cycles, the observed cognitive changes are likely to have resulted from treatment- and disease-related factors. Although MoCA cannot pinpoint the cause of deficits, over half of ICI recipients without known CNS disease showed measurable global cognitive impairment. Our study adds important insight by identifying clinical determinants associated with cognitive decline in the ICI-treated population.

Patients aged ≥65 were significantly more prone to cognitive impairment than younger patients. Aging and cancer treatments together may accelerate cognitive aging [19], highlighting the importance of cognitive monitoring in older patients. In our cohort, female patients demonstrated a greater frequency of cognitive impairment than men. This pattern may indicate sex-related differences in cognitive reserve or hormonal susceptibility [20]. Sex-related differences in symptom perception and reporting may also contribute, as females often describe higher symptom burden and poorer QoL [21]. We also showed that cognitive impairment was more frequent among patients whose education was at or below the high school level. Education is widely recognized as the primary proxy for cognitive reserve, and higher educational attainment is associated with increased resilience to brain pathology [22]. Thus, our findings support the idea that lower cognitive reserve may predispose patients to ICI-related cognitive difficulties, consistent with evidence linking reduced reserve to increased vulnerability in both cancer-related and neurodegenerative cognitive disorders [23,24]. Early cognitive screening may be particularly important in patients with limited education, who may have reduced ability to compensate for subtle impairments. Beyond education, social determinants of health and cognitive reserve may influence susceptibility to subclinical cognitive impairment detected by screening tools such as the MoCA, independent of overt neurological disease [25,26]. Importantly, this subclinical cognitive impairment should be distinguished from rare but clinically overt neurological immune-related adverse events, such as encephalitis, which typically present with acute neuropsychiatric manifestations and require urgent intervention [6].

Cognitive impairment was significantly more likely in patients receiving ICIs as second-line or later than in those receiving first-line. This result is consistent with cumulative chemotherapy exposure leading to progressive cognitive decline [27]. Cuzzubbo et al. reported that cognitive deficits in ICI-treated patients likely reflect cumulative effects of earlier chemotherapy rather than immunotherapy itself [5]. Taken together, our findings suggest that later-line cognitive changes are better explained by cumulative chemotherapy effects and more prolonged exposure to cancer and its therapies than by ICIs alone. This highlights the importance of obtaining baseline cognitive measures in patients transitioning to ICIs after chemotherapy, enabling recognition of subsequent changes.

We observed a strong correlation between functional status and cognition. These findings suggest an association between poorer ECOG performance status and worse cognitive performance, potentially reflecting greater frailty and comorbidity burden in patients with lower ECOG status [28]. From a clinical perspective, a decline in ECOG performance status during ICI therapy may be a signal for early evaluation of cognitive function. Additionally, our findings revealed that patients with two or more metastatic sites exhibited significantly greater cognitive impairment. This is consistent with evidence that metastatic cancer itself can impair cognitive function, potentially through tumor-related inflammation or paraneoplastic effects [12,29]. A higher tumor burden may elicit systemic inflammation or demand more intensive therapy, either of which can contribute to cognitive impairment [30]. Therefore, regular cognitive monitoring is warranted in ICI-treated patients with widespread metastatic disease. Notably, the pattern of metastatic spread was similar between cognitively impaired and cognitively intact patients, suggesting an association with overall disease burden rather than specific metastatic locations.

Emerging evidence suggests that ICIs may influence the CNS and cognitive function through multiple biological pathways. Experimental findings from animal models indicate that ICI therapy can trigger neuroinflammatory responses associated with cognitive and affective disturbances [31]. Consistent with these preclinical data, clinical studies have shown partial and complete intracranial responses in brain metastases from non–small cell lung cancer and melanoma following ICI treatment, suggesting that these agents can cross or modulate immune activity within the blood–brain barrier [32]. However, enhanced immune activation may lead to CNS autoimmunity, since checkpoint blockade can induce neural self-antigen cross-reactivity similar to paraneoplastic mechanisms [33]. Furthermore, immune-related hypophysitis, observed in up to 18% of patients treated with the CTLA-4 inhibitor ipilimumab [34], may cause pituitary dysfunction and hormonal imbalance, which can manifest clinically as fatigue, mood alterations, and cognitive decline [35].

From a practical standpoint, our findings support a risk-stratified framework for cognitive screening in patients receiving ICIs. Patients aged ≥65 years, female patients, those with lower educational attainment, poorer ECOG performance status (≥1), higher metastatic sites, and those treated in second- or later-line settings represent a high-risk subgroup in whom baseline and periodic cognitive screening using brief tools such as the MoCA may be particularly warranted. In contrast, screening in lower-risk patients may be guided by clinical symptoms or functional decline. Abnormal screening results should prompt evaluation of potentially reversible contributors, including mood symptoms, fatigue, sleep disturbance, and metabolic factors, and consideration of referral to neuropsychology or supportive care services when appropriate. This pragmatic, risk-based approach may facilitate early identification of vulnerable patients, support individualized management, and improve quality-of-life outcomes during ICI therapy.

This study had several limitations. First, the cross-sectional design limits causal interpretation and does not allow evaluation of cognitive changes over time. Second, cognitive status was assessed using the MoCA, a screening rather than a comprehensive neuropsychological tool, and the ≤21 cutoff may differ in validity across populations. Moreover, reliance on a single global screening instrument without domain-specific neuropsychological testing or functional outcome measures limits mechanistic insight and may overestimate cognitive impairment, particularly in patients with lower educational attainment. Third, Baseline cognitive data prior to ICI initiation were unavailable, limiting the ability to distinguish the influence of prior treatments or disease duration. Fourth, the heterogeneity of cancer types and ICI regimens within the cohort may have contributed to the observed variability in cognitive outcomes. Fifth, as the majority of patients received ICIs as monotherapy, the limited number of patients treated with concurrent or combination systemic therapies precluded a separate analysis, and regimen-specific effects on cognitive outcomes cannot be ruled out. Sixth, the total number of potentially eligible patients and the characteristics of those who did not participate were not systematically recorded, which limits assessment of possible selection bias and may affect the generalizability of prevalence estimates. Seventh, concomitant use of CNS active medications, such as antidepressants, anxiolytics, antipsychotics, or opioid analgesics, was not systematically recorded and therefore could not be accounted for in the analyses, representing a potential source of residual confounding. Additionally, standardized assessments of depression and anxiety were not available at the same time point as MoCA testing, precluding adjustment for these common psychological confounders that may influence cognitive performance. Finally, objective parameters such as imaging, inflammatory biomarkers, or actigraphy-based measures were not incorporated.

## 5. Conclusions

The present study demonstrated that cognitive impairment was frequent in patients receiving ICIs. Additionally, we found that the independent determinants of impaired cognition were older age, female sex, limited education, later-line ICI treatment, poorer ECOG performance status, and higher metastatic sites, indicating that vulnerability is primarily shaped by patient and disease characteristics rather than ICI itself. Regular assessment of cognitive function in high-risk groups, such as older adults, individuals with a lower educational background, patients with declining ECOG scores, or those with widespread metastatic disease, may improve the early identification of cognitive decline and guide supportive interventions. Given the cross-sectional design and the absence of pre-treatment baseline cognitive assessments, the findings represent associations rather than evidence of an ICI-specific causal effect. Future longitudinal cohorts are needed to initiate cognitive assessment before ICI exposure, incorporate repeated follow-up testing with concurrent mood evaluations, include appropriate comparison groups, and ultimately establish causality.

## Figures and Tables

**Table 1:** Demographic and clinical characteristics of patients according to MoCA-defined cognitive status.

Characteristics	All Patients (*n* = 189)	MoCA > 21 (*n* = 87, 46%)	MoCA ≤ 21 (*n* = 102, 54%)	*p*-Value
Age (years)				<0.001
<65	103 (54.5%)	59 (67.8%)	44 (43.1%)	
≥65	86 (45.5%)	28 (32.2%)	58 (56.9%)	
Gender				0.014
Male	138 (73%)	71 (81.6%)	67 (65.7%)	
Female	51 (27%)	16 (18.4%)	35 (34.3%)	
Education level				<0.001
≤High school (middle and high school)	116 (61.4%)	35 (40.2%)	81 (79.4%)	
≥University (undergraduate and postgraduate)	73 (38.6%)	52 (59.8%)	21 (20.6%)	
ECOG performance status				<0.001
0	69 (36.5%)	49 (56.3%)	20 (19.6%)	
1–2	120 (63.5%)	38 (43.7%)	82 (80.4%)	
Primary cancer type				0.838
NSCLC	110 (58.2%)	48(55.2%)	62 (60.8%)	
RCC	26 (13.8%)	13 (14.9%)	13 (12.7%)	
Melanoma	18 (9.5%)	8 (9.2%)	10 (9.8%)	
Others	35 (18.5%)	18 (20.7%)	17 (16.7%)	
Metastatic sites				
Lung	50 (26.5%)	20 (23%)	30 (29.4%)	0.318
Liver	41 (21.7%)	24 (27.6%)	17 (16.7%)	0.069
Bone	80 (42.3%)	33 (37.9%)	47 (46.1%)	0.259
Adrenal	27 (14.3%)	9 (10.3%)	18 (17.6%)	0.153
Comorbidities				
HT	49 (25.9%)	25 (27.8%)	24 (24.7%)	0.637
DM	19 (10.2%)	11 (12.2%)	8 (8.2%)	0.369
CVD	22 (11.8%)	11 (12.2%)	11 (11.3%)	0.852
Line of ICI				0.014
First-line	57 (30.2%)	34 (39.1%)	23 (22.5%)	
Second-line or beyond	132 (69.8%)	53 (60.9%)	79 (77.5%)	
ICI cycles at assessment				0.004
<6 cycles	87 (46%)	50 (57.5%)	37 (36.3%)	
≥6 cycles	102 (54%)	37 (42.5%)	65 (63.7%)	
Regimen type				0.455
Anti-PD-1	158 (83.6%)	70 (80.5%)	88 (86.3%)	
Anti-PD-L1	25 (13.2%)	13 (14.9%)	12 (11.8%)	
Anti-CTLA-4/combination	6 (3.2%)	4 (4.6%)	2 (1.9%)	

Abbreviations: CTLA-4: Cytotoxic T-Lymphocyte–Associated Protein 4; CVD: Cardiovascular Disease; DM: Diabetes Mellitus; ECOG: Eastern Cooperative Oncology Group; HT: Hypertension; MoCA: Montreal Cognitive Assessment; NSCLC: Non-Small Cell Lung Cancer; PD-1: Programmed Cell Death Protein 1; PD-L1: Programmed Death-Ligand 1; RCC: Renal Cell Carcinoma; SD: Standard Deviation.

**Table 2:** Univariate and multivariate logistic regression analyses of factors associated with cognitive impairment in patients receiving ICIs.

Variable	Univariate OR (95% CI)	*p*-Value	Multivariate OR (95% CI)	*p*-Value
Age ≥ 65 years	2.77 (1.53–5.04)	<0.001	2.98 (1.45–6.12)	0.003
Female sex	2.31 (1.17–4.57)	0.015	2.48 (1.09–5.67)	0.030
≤High school (middle and high school)	5.73 (3.01–10.90)	<0.001	3.10 (1.35–7.07)	0.007
Second-line or beyond	2.20 (1.17–4.15)	0.014	3.51 (1.56–7.88)	0.002
ECOG ≥ 1	5.28 (2.76–10.09)	<0.001	3.38 (1.46–7.83)	0.004
≥2 metastatic sites	2.21 (1.23–3.96)	0.008	2.85 (1.37–5.90)	0.005
ICI treatment duration ≥ 6 cycles	2.37 (1.32–4.26)	0.004	1.60 (0.77–3.30)	0.204

Abbreviations: OR: odds ratio; CI: confidence interval; ECOG: Eastern Cooperative Oncology Group; ICI: Immune Checkpoint Inhibitor.

## Data Availability

The data presented in this study are available on request from the corresponding author due to privacy and ethical restrictions related to patient confidentiality.

## Abbreviations

The following abbreviations are used in this manuscript:

CI	Confidence Interval
CNS	Central Nervous System
CRCI	Cancer-related Cognitive Impairment
CTLA-4	Cytotoxic T-Lymphocyte–Associated Protein 4
CVD	Cardiovascular Disease
DM	Diabetes Mellitus
ECOG	Eastern Cooperative Oncology Group
HT	Hypertension
ICI	Immune Checkpoint Inhibitor
IQR	Interquartile Range
MoCA	Montreal Cognitive Assessment
NSCLC	Non–Small Cell Lung Cancer
OR	Odds Ratio
PD-1	Programmed Cell Death Protein 1
PD-L1	Programmed Death-Ligand 1
QoL	Quality of Life
RCC	Renal Cell Carcinoma
SD	Standard Deviation
